# Evidence for a second ankylosing spondylitis-associated *RUNX3* regulatory polymorphism

**DOI:** 10.1136/rmdopen-2017-000628

**Published:** 2018-02-08

**Authors:** Matteo Vecellio, Adrian Cortes, Amity R Roberts, Jonathan Ellis, Carla Jayne Cohen, Julian C Knight, Matthew A Brown, Paul Bowness, Bryan Paul Wordsworth

**Affiliations:** 1 Nuffield Department of Orthopaedics, Rheumatology and Musculoskeletal Sciences, University of Oxford, Oxford, UK; 2 Nuffield Department of Orthopaedics, Rheumatology and Musculoskeletal Sciences, National Institute for Health Research Oxford Musculoskeletal Biomedical Research Unit, Oxford, UK, Oxford, UK; 3 Nuffield Department of Orthopaedics, Rheumatology and Musculoskeletal Sciences, Botnar Research Centre, Nuffield Orthopaedic Centre, National Institute for Health Research Oxford Comprehensive Biomedical Research Centre, Oxford, UK; 4 Nuffield Department of Clinical Neurosciences, Division of Clinical Neurology, John Radcliffe Hospital, University of Oxford, Oxford, UK; 5 Wellcome Trust Centre for Human Genetics, Roosevelt Drive, University of Oxford, Oxford, UK; 6 Translational Genomics Group, Institute of Health and Biomedical Innovation, School of Biomedical Sciences, Queensland University of Technology at Translational Research Institute, Queensland, Australia

**Keywords:** ankylosing spondylitis, gene polymorphism, spondyloarthritis, epidemiology

## Abstract

**Objectives:**

To explore the functions of *RUNX3* single nucleotide polymorphisms (SNPs) associated with ankylosing spondylitis (AS).

**Methods:**

Individual SNP associations were evaluated in 4230 UK cases. Their effects on transcription factor (TF) binding, transcription regulation, chromatin modifications, gene expression and gene interactions were tested by database interrogation, luciferase reporter assays, electrophoretic mobility gel shifts, chromatin immunoprecipitation and real-time PCR.

**Results:**

We confirmed the independent association of AS with *rs4265380*, which was robust (P=4.7×10^−6^) to conditioning on another nearby AS-associated *RUNX3* SNP (*rs4648889*). A *RUNX3* haplotype incorporating both SNPs was strongly associated with AS (OR 6.2; 95% CI 3.1 to 13.2, P=1.4×10^−8^). In a large UK cohort, *rs4265380* is associated with leucocyte counts (including monocytes). *RUNX3* expression is lower in AS peripheral blood mononuclear cells than healthy controls (P<0.002), independent of *rs4265380* genotype. Enhancer function for this *RUNX3* region was suggested by increased luciferase activity (approximately tenfold; P=0.005) for reporter constructs containing *rs4265380*. In monocytes, there was differential allelic binding of nuclear protein extracts to a 50 bp DNA probe containing *rs4265380* that was strongly augmented by lipopolysaccharide activation. TF binding also included the histone modifier p300. There was enrichment for histone modifications associated with active enhancer elements (H3K27Ac and H3K79Me2) that may be allele dependent. Hi-C database interrogation showed chromosome interactions of RUNX3 bait with the nearby RP4-799D16.1 lincRNA.

**Conclusions:**

The association of AS with this *RUNX3* regulatory region involves at least two SNPs apparently operating in different cell types. Monocytes may be potential therapeutic targets in AS.

**Key messages**

**What is already known about this subject?**

- From genome-wide association studies, it appears that more than 100 genes are involved in susceptibility to ankylosing spondylitis (AS). One of these is *RUNX3*, a transcription factor involved in the maturation of several immune cell types.
- Several single nucleotide polymorphisms (SNPs) upstream the promoter of *RUNX3*, which are strongly associated with AS lie in a putative genomic regulatory region. One of these SNPs, *rs4648889,* appears to be operative particularly in CD8+ T cells.

**What does this study adds?**

- We report potential functional effects from a second independently associated SNP in this *RUNX3* region (*rs4265380)* that may be restricted to monocytes rather than T cells. The independent associations of AS with these two SNPS and the likelihood that they have functional effects in two different cell types suggest that both CD8+ T cells and monocytes could be therapeutic targets in AS. Understanding the functions of this *RUNX3* could provide new insights into the pathogenesis of AS.

**How might this impact on clinical practice?**

- By investigating the AS-associated mechanisms controlling RUNX3 transcription and the regulatory effects of RUNX3 itself on downstream gene expression, we expect to identify pathways that include credible therapeutic targets for future drug development.

## Introduction

Although genetic predisposition to ankylosing spondylitis (AS) is strongly associated with the human leukocyte antigen (HLA)-B27 immune response gene genome-wide association studies (GWAS) have clearly shown that it is polygenic.^1 2^ Several robust associations have been identified with single nucleotide polymorphisms (SNPs) in coding sequences, including other genes in the major histocompatibility complex, *ERAP1* and *IL23R*.^1–6^ However, as in other complex polygenic disorders, AS-associated SNPs are more typically located in non-coding regions of the genome, such as the *IL23R-IL12RB2* intergenic region, where they may influence gene expression.^7–10^

Convincing associations between AS and SNPs upstream of *RUNX3* (Runt-related transcription factor 3) have previously been demonstrated by GWAS.^4 11 12^ Subsequently, the International Genetics of AS (IGAS) Immunochip study demonstrated that 22 SNPs in a ~15 kb linkage disequilibrium (LD) block upstream of the *RUNX3* promoter are strongly associated with AS (lead SNP *rs6600247*: P=1.3×10^−14^).^1^ We have previously shown that this LD block contains a putative regulatory region; further, we demonstrated that the risk allele at *rs4648889* within this block reduces recruitment of the interferon regulatory factor 4 (IRF4) transcription factor (TF), leading to reduced *RUNX3* expression in CD8+ T cells in an allele-dependent fashion.^11^ RUNX3 not only plays a key role in the development of CD8+ T cells^13 14^ but also has plausible roles in the development of many other immune cell types, including the differentiation, activity and function of monocytes.^15–17^ Both CD8+ T cells and monocytes have been shown to have a role in AS pathogenesis.^18 19^

Here, we present evidence for the complexity of both the molecular and cellular associations of *RUNX3* with AS. Having previously established the strong association of AS with *rs4648889* in this region (P=1.3×10^−14^),^11^ we now confirm the presence of a second independent association with a second SNP—*rs4265380.* We characterise some of the functional effects of this SNP and conclude that these two adjacent but independent signals probably exert their effects in different cell types.

## Materials and methods

### Genotyping

Historical genotype data from the UK subset of cases included in the AS Immunochip study (n=4230) and ethnically matched healthy controls (n=9200) were used in the association studies.^1^ Where necessary for cases used in the functional studies, DNA was extracted using the Qiagen AllPrep DNA/RNA Mini Kit (Qiagen Ltd, Manchester, UK) and genotyped for *rs4265380* using TaqMan SNP assay (Life Technologies, Paisley, UK).

### AS cases used in functional studies

Following written informed consent (COREC 06/Q1606/139 and OXREC B 07/Q1605/35), venous blood samples were obtained from 17 cases fulfilling either the modified New York criteria for AS or the  Assessment in SpondyloArthritis international Society  (ASAS) imaging criteria for axial spondyloarthropathy.^20 21^ Their average age was 45 years (range 24–64); medications included non-steroidal anti-inflammatory drugs (n=13) and sulfasalazine (n=2); all cases were biologic therapy naive. Disease activity assessed by the Bath AS Disease Activity Index (BASDAI)^22^ and C-reactive protein (CRP) showed substantial variation (mean BASDAI=3.2, range 1.2–6.6; mean CRP=6.1 mg/L, range 0.2–19).

### Cell transfection and luciferase reporter assay

Cloning of the 250 bp sequence flanking *rs4265380* was performed as previously described.^11^ The constructs were efficiently transfected into human embryonic kidney HEK-293T cell line using Lipofectin (Life Technologies) according to manufacturer’s instructions. Transfection was carried out for 48 hours. The Dual-Luciferase assay reporter system (Promega, Madison, USA) was used to evaluate luciferase activity. Firefly luciferase activity was normalised relative to Renilla luciferase activity for each transfection and calculated as fold increase over pGL4.23[luc2/minP].

### CD14+ monocyte isolation and stimulation

CD14+ monocytes were isolated from peripheral blood mononuclear cells (PBMCs) using a monocyte isolation kit (Miltenyi, Bisley, Surrey, UK). Monocytes were resuspended at 1×10^6^/mL in prewarmed Roswell Park Memorial Institute medium supplemented with 10% fetal bovine serum, penicillin/streptomycin and L-glutamine and rested overnight.

Monocytes (4×10^5^) were stimulated for 24 hours with or without ultrapure lipopolysaccharide (LPS  20 ng/mL; Invivogen, San Diego, USA) before harvesting for experiments.

### Electrophoretic mobility gel shift assay (EMSA)

A 50-bp DNA probe including *rs4265380* was incubated with nuclear extracts obtained either from primary CD14+ monocytes stimulated with LPS for 24 hours or a monocyte cell line from histiocytic lymphoma (U937) stimulated with phorbol-12-myristate-13-acetate (PMA). Nuclear extracts from monocytes and U937 cells were prepared using the Thermo Scientific NE-PER Nuclear and Cytoplasmic Extraction kit (Thermo Scientific, Waltham, USA). EMSA was performed using LightShift Chemiluminescent EMSA Kit (Thermo Scientific) with no modifications. Single-stranded biotinylated oligonucleotides (prepared with biotin 3′ end DNA labelling kit) were mixed and annealed at room temperature for 1 hour. Five micrograms of nuclear extract and 0.5 ng biotin labelled double-stranded oligonucleotides (50 bp fragment; Eurofins, Wolverhampton, UK) were used in each experiment. Unlabelled probes in 100-fold excess were used as competitor.

The sequences of the synthetic single-stranded oligonucleotides are listed below:

C* s (sense): 5′-CTCCATGACGCAATTTGGGCTCCGTTATGAGTCAGCTCAAGTAA-3′;

T* s: 5′-CTCCATGACGCAATTTGGGCTCTGTTATGAGTCAGCTCAAGTAA-3′;

C* as (antisense): 5′-TTACTTGAGCTGACTCATAACGGAGCCCAAATTGCGTCATGGAG-3′;

T* as: 5′-TTACTTGAGCTGACTCATAACAGAGCCCAAATTGCGTCATGGAG-3′.

(Underlined base highlights the position of *rs4265380*).

### Chromatin immunoprecipitation (ChIP)-qPCR

ChIP was performed using the Low Cell# ChIP kit (catalogue ID C01010072, Diagenode, Liege, Belgium) as previously described.^11^ For each ChIP, 3×10^5^ monocytes, either unstimulated or LPS stimulated for 24 hours, were used. DNA was isolated with the Low Cell# ChIP kit (Diagenode) and qPCR performed on immune complex-associated DNA using allele-specific primers for *rs4265380* (WT sense primer: 5′-TGACGCAATTTGGGCTAC-3′; AS-risk sense primer: 5′-TGACGCAATTTGGGCTAT-3′; common antisense primer: 5′-CCAGCTGTGTCATTCTCCAA-3′), detected with SYBR Green (Qiagen Ltd) on ABI ViiA 7 PCR device (Applied Biosystems, Paisley, UK).

Relative occupancy was calculated as a ratio of specific signal over background: % input (specific loci)/% input (background loci). The following ChIP grade antibodies were used: p300 ((C-20), SC585X, Santa Cruz Biotechnology, Dallas, USA), H3K4Me1 (Diagenode, Mab-150–050), H3K79Me2 (Diagenode, C15410051), H3K27Ac (Diagenode, Mab-184–050) and IgG (Diagenode, K02041008).

### Quantitative real-time RT-PCR

RNA was isolated with TRIzol (Invitrogen, Paisley, UK) and reverse transcribed with Superscript III (Invitrogen) to synthesise cDNA as previously described.^11^ The specific primers were: RUNX3 sense (s): 5′-ACTCAG CAC CAC AAG CCA CT-3′; RUNX3 antisense (as): 5′-GTC GGA GAA TGG GTT CAG TT-3′; RP4-799D16.1 s: 5′-CAG ATG GCA TTT GGG ATC CA-3′’; RP4-799D16.1 as: 5′-TTC CTG TGT TGG CAT CTG AC-3′.

PCRs were performed in triplicate and the 2−ΔCt method was used to calculate the expression of each gene. All values were normalised to β-actin (ID Assay qHsaCED0036269, Bio-Rad Laboratories, UK).

miRNA quantitative real-time RT-PCR miRNeasy Mini Kit (Qiagen Ltd) was used to isolate total RNA, according to the manufacturer’s instructions. Ten nanograms of RNA was reverse transcribed and quantified by TaqMan real-time PCR using the TaqMan microRNA Assay (hsa-miR-4425, assay ID: 462954_mat, Applied Biosystems). RNU6 was used as endogenous control (U6 snRNA, assay ID: 001973). For detection, we used a vii7 PCR machine (Applied Biosystems).

### Bioinformatics

We used publicly available data from the ENCODE (https://genome.ucsc.edu/ENCODE/) and Roadmap Epigenomics (http://epigenomegateway.wustl.edu/browser/roadmap/) projects and previously published GWAS association data^1^ to identify regulatory elements, histone modifications and TF binding sites relevant to AS in the *RUNX3* region.^23 24^ Potential chromatin interactions were evaluated using data generated by the International Human Epigenome Consortium (https://www.chicp.org/chicp).^25^ *RUNX3* transcription in AS cases and controls was evaluated from historical data derived from RNA-seq in PBMCs from 72 AS cases and 62 healthy controls.^19^

### Statistical analysis

Association analysis was performed using the logistic regression function in PLINK,^26^ V.1.07, accounting for population structure with 10 principal components as covariates in the regression analysis and conditioning on the *RUNX3* SNP *rs4648889*.^11^ Haplotypes were inferred by phasing using the SHAPEIT tool^27^ and all SNPs genotyped in the locus (chr1: 23038732–27660734). Haplotype counts in cases and controls were compared using the χ^2^ test and measured relative to the most common haplotype found in the control samples. One-way analysis of variance and two-tailed Student’s t*-*test were used to determine statistical significance using the GraphPad Prism software (V.7.03) package.

## Results

### Identification of *rs4265380* as a putative candidate causal variant in monocytes

**Figure 1:**
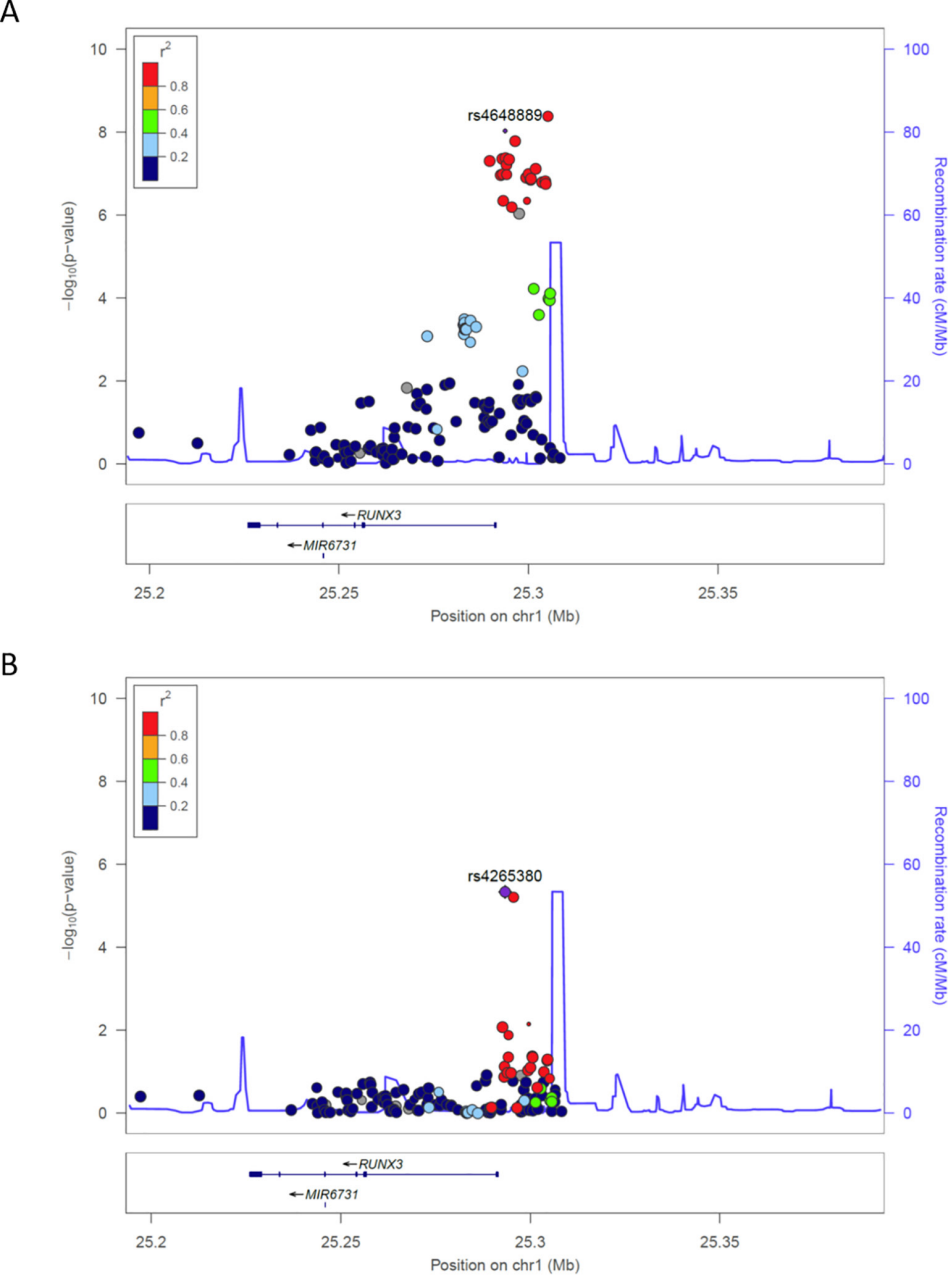
Ankylosing spondylitis susceptibility associations with *RUNX3* in UK cohort. (A) Unconditional association showing *rs4648889* primary signal. (B) The plot shows association over *RUNX3* after conditioning for SNP *rs4648889*, retaining positive association for *rs4265380*.

**Figure 2:**
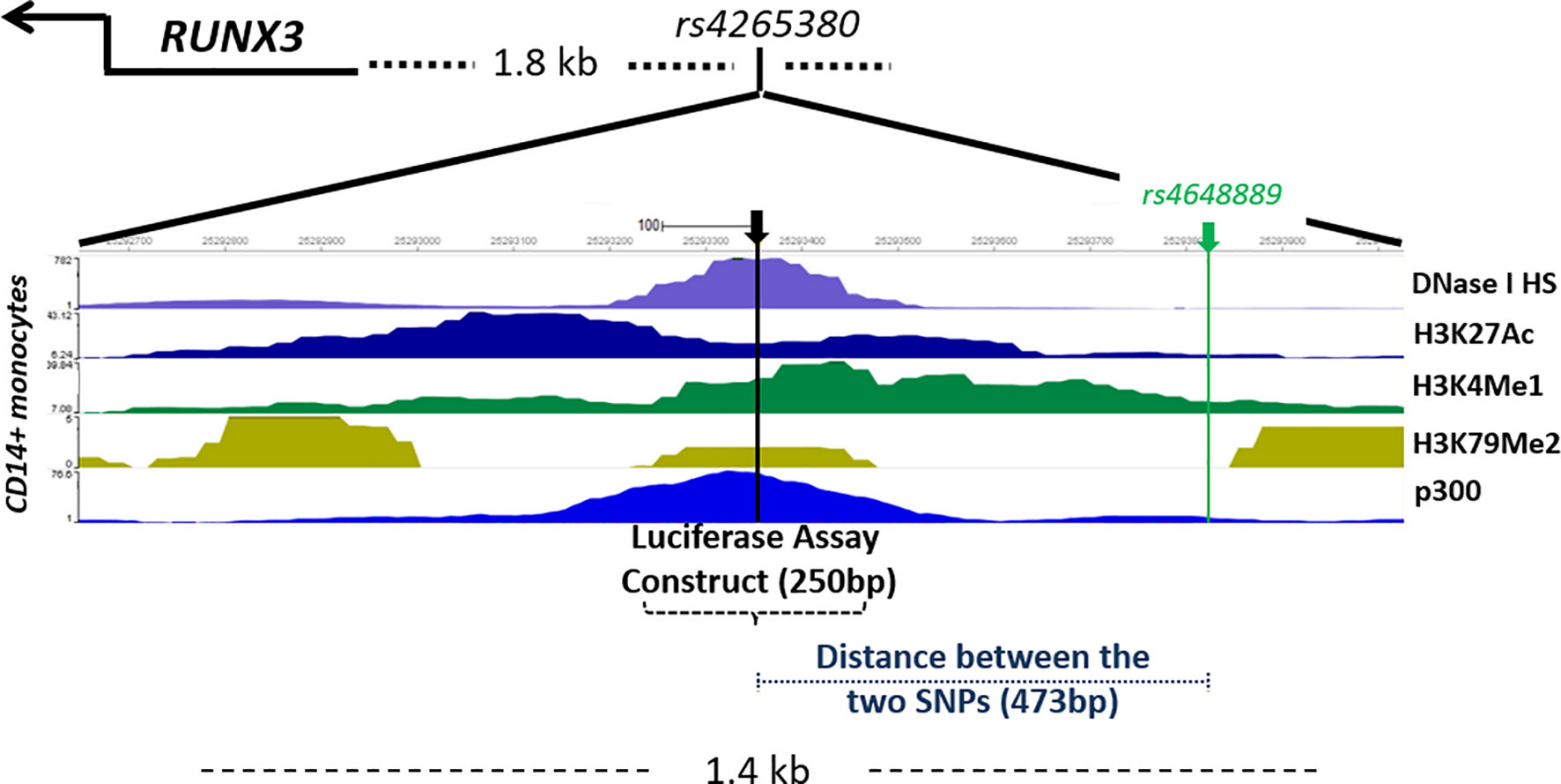
Genetic mapping and epigenetic landscape of *RUNX3* locus overlapping *rs4265380*. The region including *rs4265380* (GRCh37/hg19 human genome build; chr1: 25292650–25294000) is shown comprehensive of single nucleotide polymorphism (SNP) location, *RUNX3* gene position and chromatin state. DNase I hypersensitivity and histone modification chromatin immunoprecipitation sequencing (ChIP-seq) data (Roadmap EpiGenome Browser V.1.19) including H3K4Me1, H3K27Ac and H3K79Me2 for CD14+ monocytes are shown. ChIP-seq for p300 transcription factor is shown for the available lymphoblastoid cell line GM12878.^23 24^ The position of *rs4648889* SNP and the location of the 250-bp construct used in luciferase assay are also shown.

The region upstream of *RUNX3* contains potentially important regulatory sequences. However, the strongest association with AS in this region is with *rs6600247 (*P*=*4.23×10^−9^) that does not overlap with obvious functionally active sites. We therefore investigated SNPs in strong LD with *rs6600247* that lie in a region associated with open chromatin for evidence of regulatory activity; these include *rs4648889* (unconditional analysis P=9.5×10^−9^, figure 1A) that is functionally active in CD8+ T cells. Of the 22 *RUNX3* SNPs previously associated with AS in the IGAS Immunochip study at P≤5×10^−7^, *rs4265380* (P=4.58×10^−7^) was the most strongly associated (with the ‘C’ allele) after conditioning on *rs6600247* (lead IGAS SNP) or *rs4648889* (figure 1B). Interrogation of the publicly available ENCODE and Roadmap databases (figure 2) shows that *rs4265380* is close to potential regulatory sequences active in CD14+ monocytes that include: (1) DNase I hypersensitivity sites; (2) H3K4Me1, H3K27Ac and H3K79Me2 histone modifications that are associated with regulatory regions and replication initiation sites and (3) ChIP sequencing peaks for TF binding, particularly p300 (a transcriptional activator and histone reader). This region, therefore, appears to contain a potential regulatory element upstream of RUNX3 specifically active in monocytes, in which the C allele at *rs4265380* increases the risk of AS.

### Haplotype analysis showed the presence of a specific AS-associated haplotype

We included the two SNPs—*rs4265380* and *rs4648889*—that were both independently associated with AS in a haplotype analysis (table 1). Both SNPs were formally genotyped rather than imputed. The CA haplotype was associated with a particularly high risk for AS (OR 6.2; 95% CI 3.1 to 13.2, P=1.4×10^−8^) compared with the most common CG haplotype. The OR of CA relative to TA is 5.35 (P=2.7×10^−7^).

**Table 1:** Haplotype analysis based on the UK subset described in the International Genetics of Ankylosing Spondylitis Consortium Immunochip study showing the presence of four distinct *RUNX3* haplotypes including *rs4265380* and *rs4648889*. The CA haplotype has the highest risk for ankylosing spondylitis relative to the common reference CG haplotype

rs4265380 rs4648889	Counts in cases	Counts in controls	Frequency in cases	Frequency in controls	P value	OR
CG	3882	9590	0.46	0.49		
TA	4477	9589	0.53	0.49	5.7×10^–8^	1.15 (1.1–1.2)
TG	5	31	6×10^–4^	0.002	6.3×10^–2^	0.4 (0.12–1.03)
CA	30	12	0.004	6×10^–4^	1.4×10^–8^	6.2 (3.1–13.2)

### *rs4265380* affects monocyte cell counts

We interrogated the Roslin Gene Atlas (http://geneatlas.roslin.ed.ac.uk/), a public database including more than 400 000 individuals (from the UK Biobank), to investigate a plausible role for *rs4265380* associations with leucocyte cell counts. The results for *rs4265380* showed a significant reduction in monocyte (P=0.006), neutrophil (P=6.4×10^−7^) and eosinophil (P=8.3×10^−7^) counts associated with the C allele. No association was apparent for lymphocyte counts (P=0.2), consistent with our hypothesis that *rs4265380* is acting specifically in monocytes.

### *RUNX3* expression is downregulated in AS cases compared with healthy controls

The historical data on *RUNX3* mRNA in PBMCs measured by RNA-seq showed significantly reduced expression (P<0.002) in AS cases compared with healthy controls. When these results were stratified on *rs4265380*, there was no apparent influence of this SNP on expression levels (online Supplementary figure 1A).

10.1136/rmdopen-2017-000628.supp1 Supplementary file 1

### The region spanning *rs4265380* shows regulatory activity in a reporter gene assay

**Figure 3:**
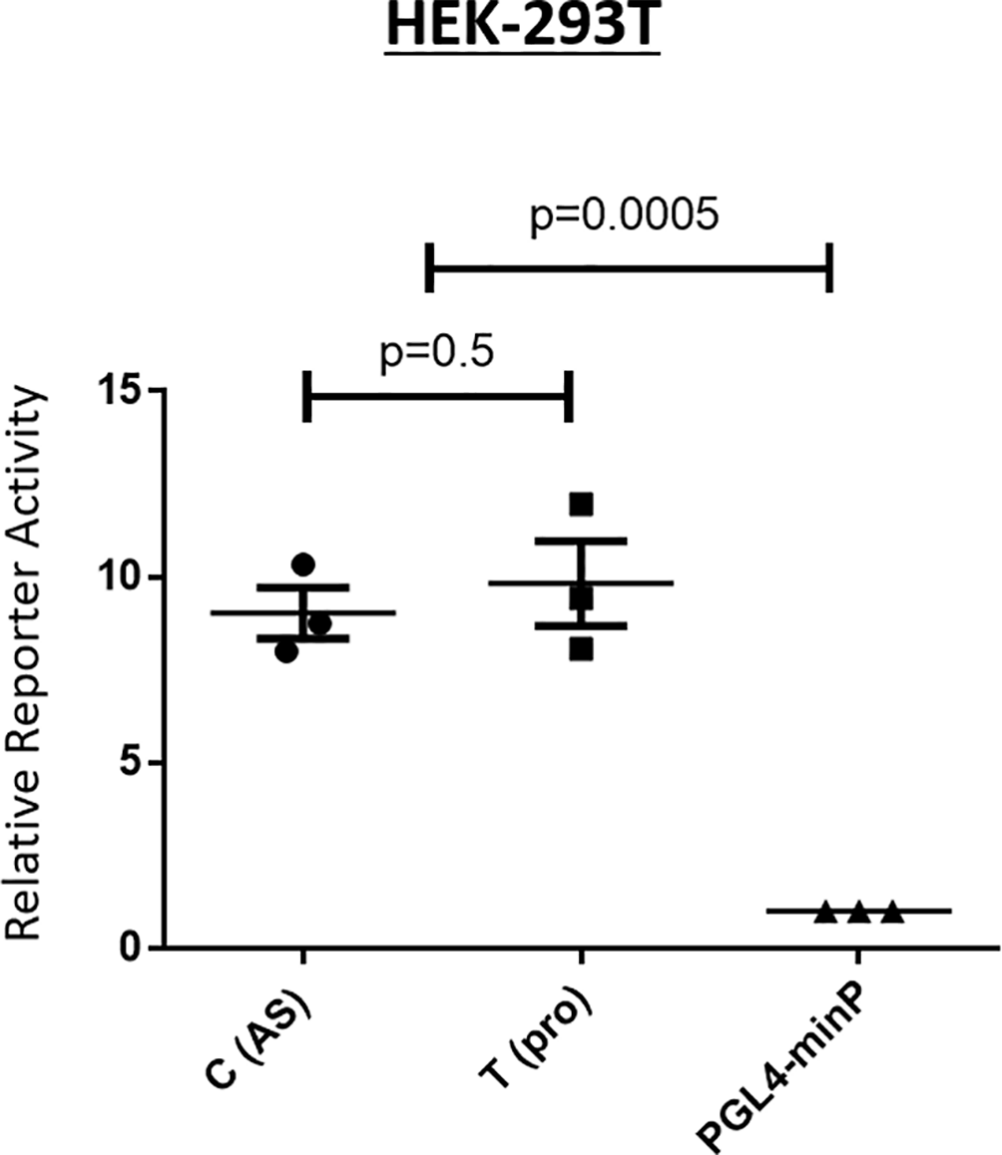
The genomic region spanning *rs4265380* shows enhancer activity. The reporter activity of *rs4265380* compared with minimal promoter construct (minP) and normalised to Renilla internal control was measured by luciferase assays on HEK293T (n=3). The values of relative luciferase activity are expressed as mean±SEM. (P=0.0005, Student’s t-test). AS, ankylosing spondylitis.

We performed luciferase reporter assays to evaluate possible enhancer activity of the region spanning *rs4265380*. Reporter constructs including the 250-bp DNA sequence spanning *rs4265380* increased luciferase activity approximately 10-fold (P=0.0005) compared with the minimal promoter alone, which was consistent with its having enhancer activity. Since there was no apparent difference between constructs containing either the *rs4265380* C (risk) or T (protective) allele in this system (figure 3), we chose to investigate possible allelic differences by other methods.

### *rs4265380* shows differential allelic binding of monocyte nuclear extract that involves the p300 transcription activator

**Figure 4:**
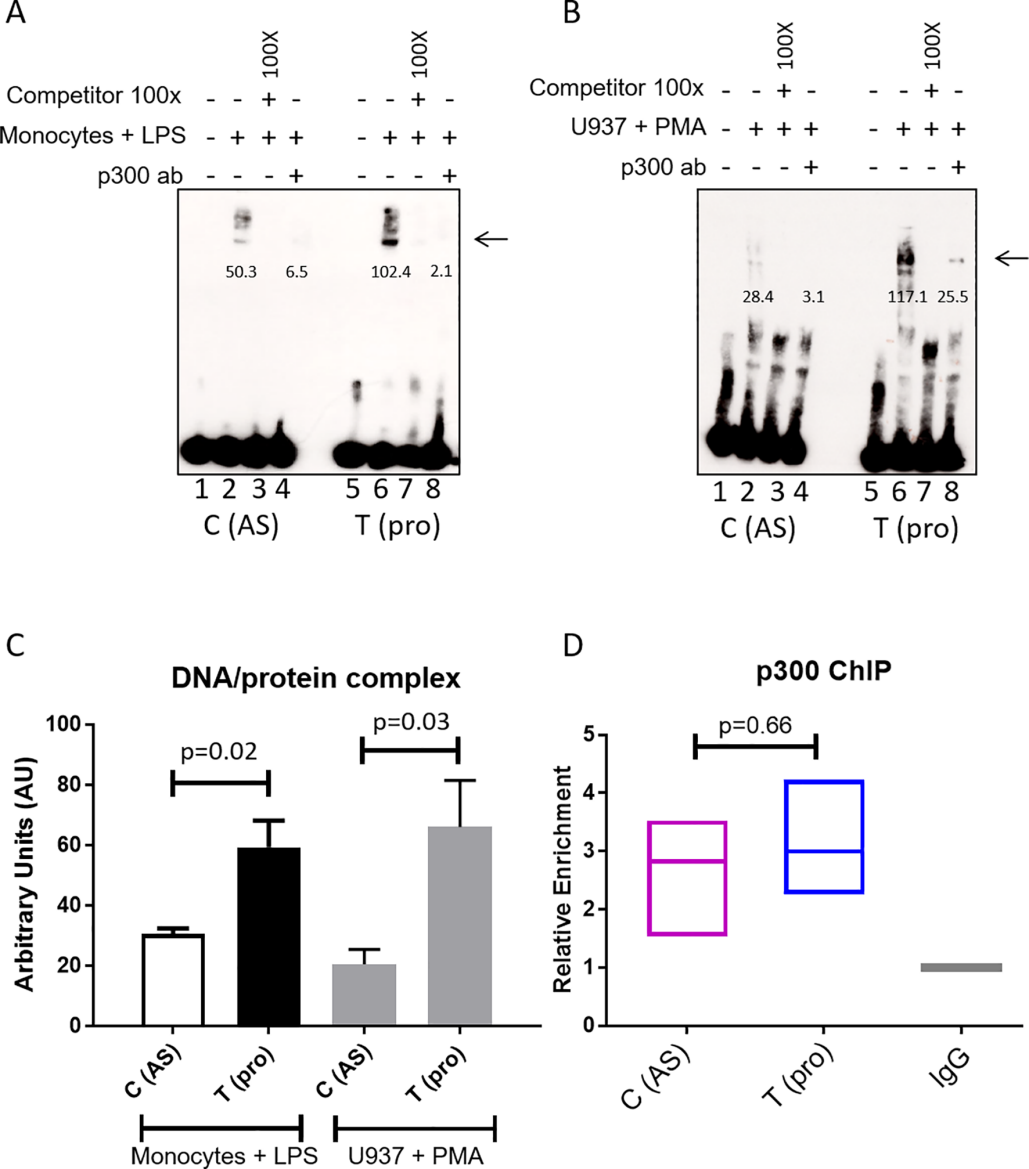
rs4265380 alters protein/DNA complex formation with the involvement of p300. (A and B) Representative EMSA showing differential nuclear extract binding (complex as indicated by arrow) after addition of stimulated primary monocytes (A) and U937 cells (B) (lanes 2 and 6). The 100-fold excess of unlabelled probes has been used as competitor (lanes 3 and 7). p300 involvement was assessed by adding p300 antibody (lanes 4 and 8). Arrows indicate the presence of DNA/nuclear extract complex. Numbers below the band represent the pixel intensity measured with Image J. (C) Quantification of the DNA/protein complex of six independent EMSAs performed on both stimulated monocytes and U937 cells (P=0.019 and P=0.031, respectively, Student’s t-test). (D) Relative enrichment of p300 was assessed with chromatin immunoprecipitation experiments (n=3) on rs4265380 CT heterozygote CD14+ monocytes (after 24 hours stimulation with LPS). AS, ankylosing spondylitis; EMSA, electrophoretic mobility shift assay; LPS, lipopolysaccharide; PMA, phorbol-12-myristate-13-acetate.

We performed EMSAs to evaluate the impact of the *rs4264380* polymorphism on DNA/TF binding. A major protein/DNA complex was seen with both monocyte (figure 4A arrowed) and U937 nuclear extracts (figure 4B arrowed). Little difference in binding was seen between the two *rs4265380* alleles in unstimulated cells (online Supplementary figure 2A arrowed), but when stimulated monocyte and U937 cell nuclear extracts were used, a much stronger band was seen for the T (protective) allele. Successful competition with 100-fold excess of unlabelled probe confirmed the specificity of these experiments (figure 4A and B and online Supplementary figure 2A). Quantification of the DNA/protein complex is shown in figure 4C (n=6 experiments, monocytes+LPS: P=0.02; U937+PMA: P=0.03). Preincubation with p300 antibody also greatly reduced the binding of nuclear extracts from both stimulated monocyte and U937 cells, suggesting that p300 is included in the complex (figure 4A and B). We conducted ChIP-qPCR (n=3) on LPS-stimulated CD14+ monocytes to assess the enrichment of p300, which was found to be similar for both alleles (figure 4D). Additional experiments performed with CD8+ T cells and stimulated Jurkat nuclear extracts (online Supplementary figure 2A) showed no differential binding thereby confirming that the effect of *rs4265380* appears to be restricted to a monocyte-like milieu.

### Other *cis*-interactions with the *RUNX3* locus

**Figure 5:**
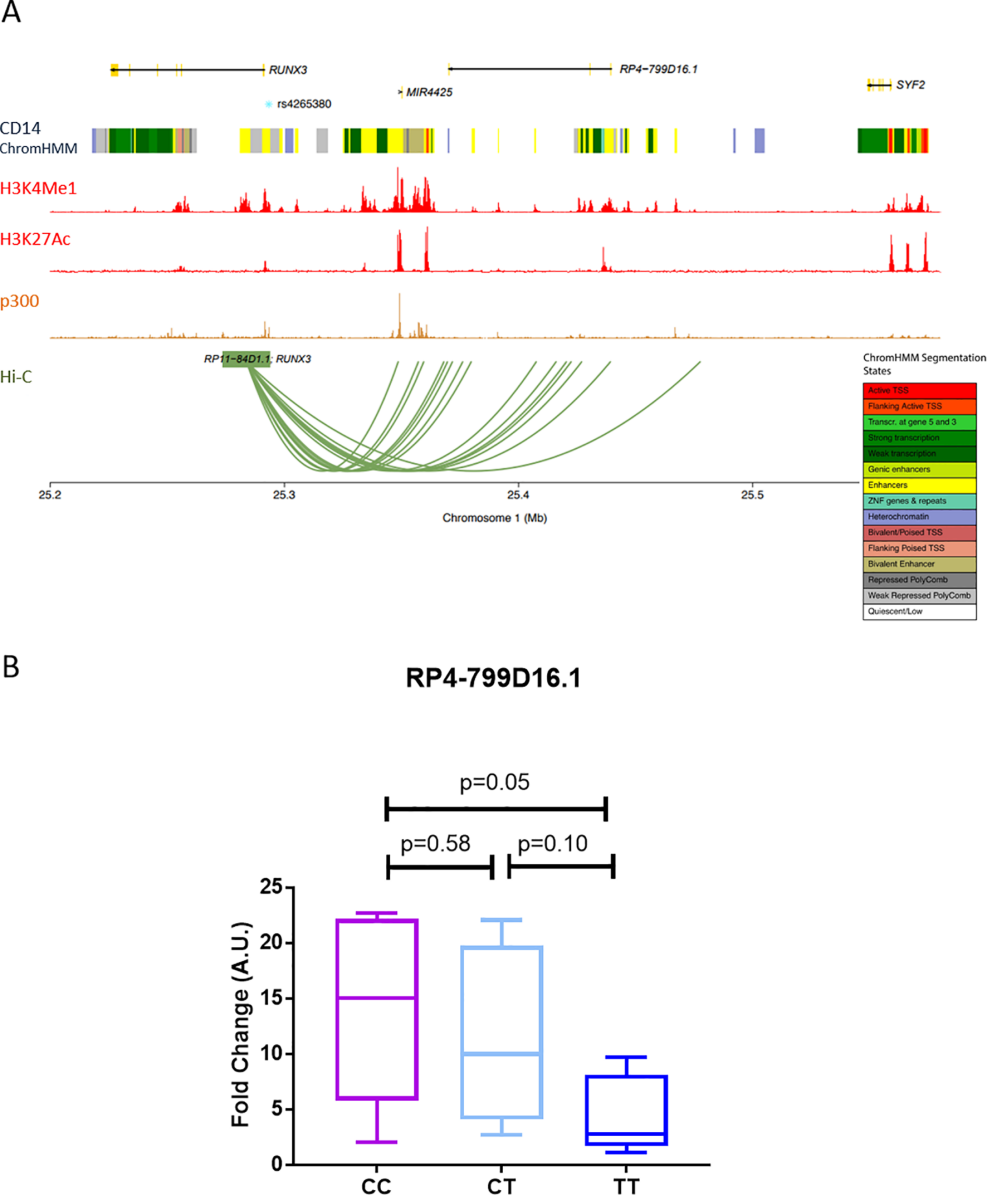
rs4265380 interacts with RP4-799D16.1 (A) Hi-C plot showing the interaction of the RUNX3 bait with different regions within chromosome 1 (with score above 5, in CD14+ monocytes). The location of genes, ENCODE CD14+ monocytes chromatin states, ENCODE histone modification and p300 transcription factor chromatin immunoprecipitation sequencing experiments and rs4265380 position are also shown. The plot has been generated through the International Human Epigenome Consortium database (https://www.chicp.org/chicp)^18^. (B) RP4-799D16.1 transcript was measured in primary CD14+ monocytes from 16 patients with ankylosing spondylitis with rs4265380 different genotypes (CC, CT and TT). P values were determined using two-tailed Student’s t-test.

The *RUNX3* locus lies close to two non-coding RNAs (miR-4425 and RP4-799D16.1 lincRNA) and also the *SYF2* locus, which is 240 kb upstream of the *RUNX3* promoter. By interrogating the Capture Hi-C plotter database (https://www.chicp.org/chicp),^25^ we observed interactions between *RUNX3* bait (overlapping *rs4265380*) and other regions of chromosome 1, specifically with RP4-799D16.1 and miR4425, but not with the more distant *SYF2* locus (figure 5A). Expression of RP4-799D16.1, a lincRNA with unknown function, showed a significant (P=0.048) increase in LPS-stimulated CD14+ monocytes from AS cases with the CC (AS-risk genotype) compared with the TT genotype (figure 5B). miR-4425 was not detected (data not shown). There was no discernible effect on the expression of *SYF2* (data not shown). *rs4265380* AS-risk C allele alters enrichment for specific histone marks in stimulated monocytes.

We assessed the enrichment of specific histone marks associated with active chromatin in this region by ChIP-qPCR (n=3) on LPS-stimulated CD14+ monocytes. There was relative enrichment for H3K79Me2 (average ~3.1-fold, P=0.04) and H3K27Ac (average ~4.6-fold, P=0.03) in the presence of the C (risk) allele (online Supplementary figure 3A), while H3K4Me1 was unchanged. No significant allelic difference was seen in unstimulated monocytes (data not shown).

## Discussion

Finding mechanistic explanations for the multiple genetic associations of common diseases, such as AS, is challenging. A small minority of such SNP associations alter protein function that have yielded insights with potential therapeutic implications. Thus, the AS association with *rs11209026*, encoding an R381Q loss of signalling function dimorphism in the cytoplasmic tail of the IL23 receptor, highlights the importance of the IL23/IL17 axis in AS.^6 28^ This influenced the successful introduction of anti-IL17A biologics for the treatment of AS.^29^ However, most GWAS ‘hits’ lie in non-coding regions of the genome with little or no immediately obvious functional significance.^9 30^In a cross-disease analysis, Ellinghaus *et al* identified 113 possible non-HLA-B27 AS associated loci, of which only 13 were exonic.^31^ Furthermore, of the 39 AS associations in this study, only one key SNP was in an exon.^19^ It seems likely that at least some of these associated SNPs exert regulatory functions either on individual genes or gene networks but unravelling these relatively subtle effects will be difficult,^8 32^ and the *RUNX3* locus is unlikely to be an exception.

Previously, we showed that *rs4648889,* an AS-associated SNP in this regulatory region upstream of *RUNX3,* influenced TF binding and gene transcription in CD8+ T cells.^11^ Here, we have demonstrated additional complexity in the *RUNX3* association with AS. Although *rs4265380* is only ~500 bp from *rs4648889*, not only is its association with AS independent of other AS-associated SNPs in this region but also it likely acts in a different cell type. DNA sequences in the vicinity of *rs4265380* show regulatory activity (demonstrated by luciferase reporter activity in vitro) and allele-specific effects on TF binding and histone modifications that could potentially alter gene expression. The relevant TF/DNA complexes include p300, a histone acetyltransferase associated with open chromatin that is involved in transcriptional activation.^33 34^ Such coactivators may not bind directly to DNA in a sequence-specific manner, but can function as histone modifiers (eg, histone acetyltransferases) or chromatin remodellers as part of the TF/DNA complex.^35^

Our data suggest that the *rs4265380* association with AS is probably explained by operational differences in monocytes rather than CD8+ T cells. First, interrogation of epigenetic databases shows relevant areas of open chromatin, TF binding and enrichment for chromatin markers of active transcription limited to monocytes. Second, the allele-specific effects on TF binding appear to be restricted to nuclear extracts from monocytes (particularly after stimulation with LPS).

In the EMSA studies, binding of p300 to *rs4265380* was allele specific, but ChIP in heterozygous C/T monocytes from AS cases indicates that p300 actually binds to both *rs4265380* alleles in the context of native chromatin. The allelic difference we observed in EMSA is unlikely to be driven by p300 alone but more likely by a multicomponent TF complex recruiting the relevant transcriptional machinery. It is not yet clear whether these findings translate to influences on the expression of nearby genes, including *RUNX3*. The complexity of the *RUNX3* associations and their multiple potential physical chromosomal interactions is further highlighted by the possible interaction of RUNX3 bait (including *rs4265380*) with a neighbouring non-coding RNA (RP4-799D16.1 lincRNA) of unknown function (see Hi-C plot in figure 5).

The observation that PBMCs from AS cases appear to have reduced *RUNX3* expression may be relevant to the pathogenesis of AS. However, it does not correlate with the *RUNX3* genotypes that we have described here. We cannot exclude a disease-specific effect on *RUNX3* expression or an effect arising elsewhere in the genome. In contrast, analysis of the UK Biobank data on the Roslin Gene Atlas database showed a significant association of *rs4265380* with monocyte, neutrophil and eosinophil counts that might be relevant to an immune-mediated disease like AS. Further evaluation of these observations in well-defined cell types from AS cases seems justified.

We have also identified an extended *RUNX3* AS-risk haplotype that includes the independent risk alleles for *rs4265380* and *rs4648889*.^11^ The finding of two independent AS-associated SNPs in such close proximity possibly operating in different cell types highlights a role for *RUNX3* in AS and perhaps suggests that both CD8+ T cells and monocytes may be involved in the pathogenesis of AS. At least in principle, both these cell types could be targeted in the treatment of AS, analogous to the use of anti-CD20 monoclonal antibody therapy targeting B cells in the treatment of rheumatoid arthritis.^36^

In summary, we have identified a regulatory region upstream of *RUNX3* that could be relevant to the pathogenesis of AS. The association of this region with AS is complex, including at least two neighbouring SNPs. There is some evidence that these have distinct cellular functional profiles, possibly indicative of pathogenic roles for both CD8+ T cells (shown previously) and monocytes in AS. However, substantial further work will be necessary to confirm this.
