# Supplementary material for: Evidence for a second ankylosing spondylitis-associated RUNX3 regulatory polymorphism
Source: RMD Open. 2018 Feb 8;4(1):e000628. doi: 10.1136/rmdopen-2017-000628 (PMC5845418; doi:10.1136/rmdopen-2017-000628)
Supplement: Supplementary file 1 [file rmdopen-2017-000628supp001.pdf]

Supplementary Figure 1

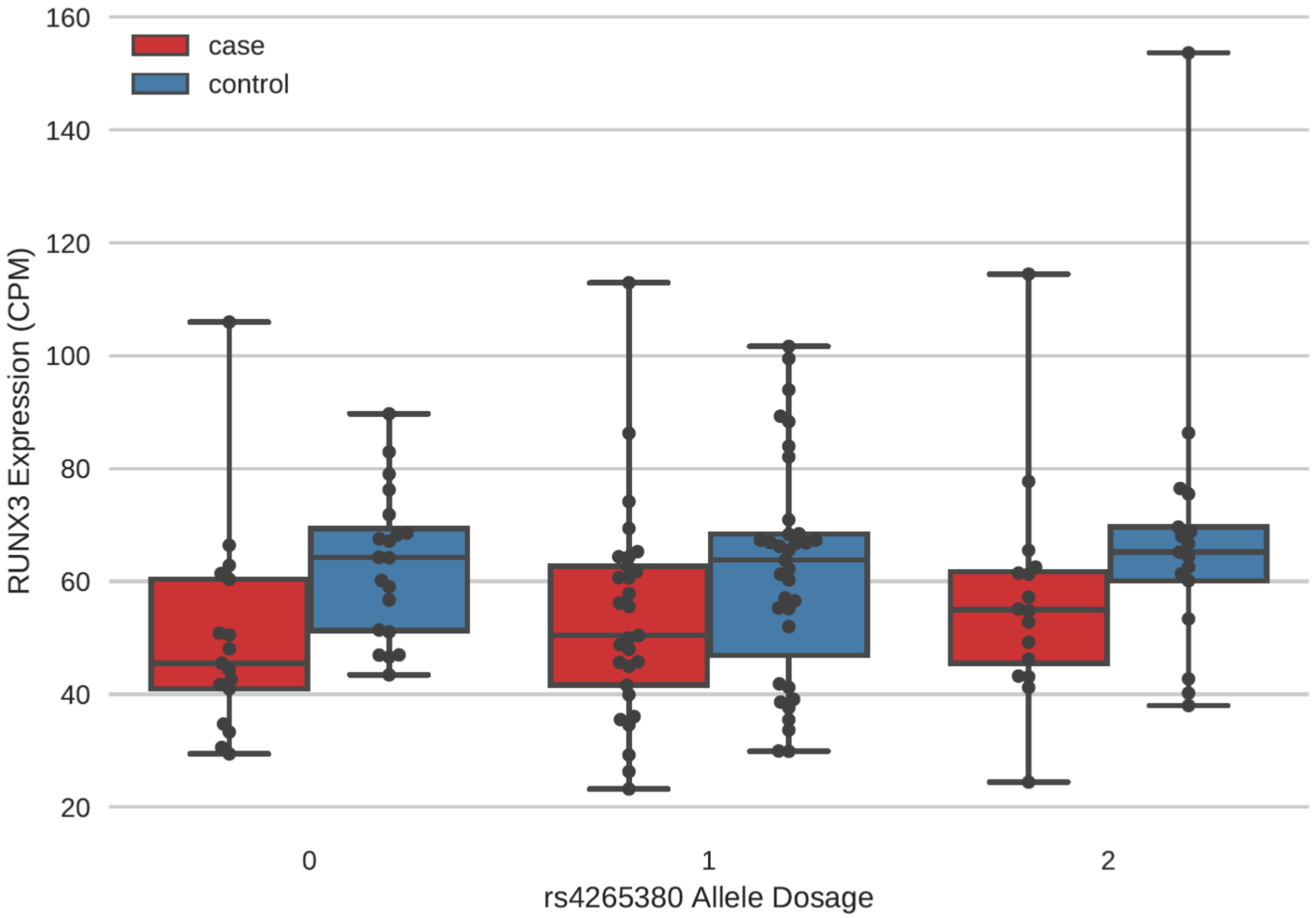

Supplementary Figure 2

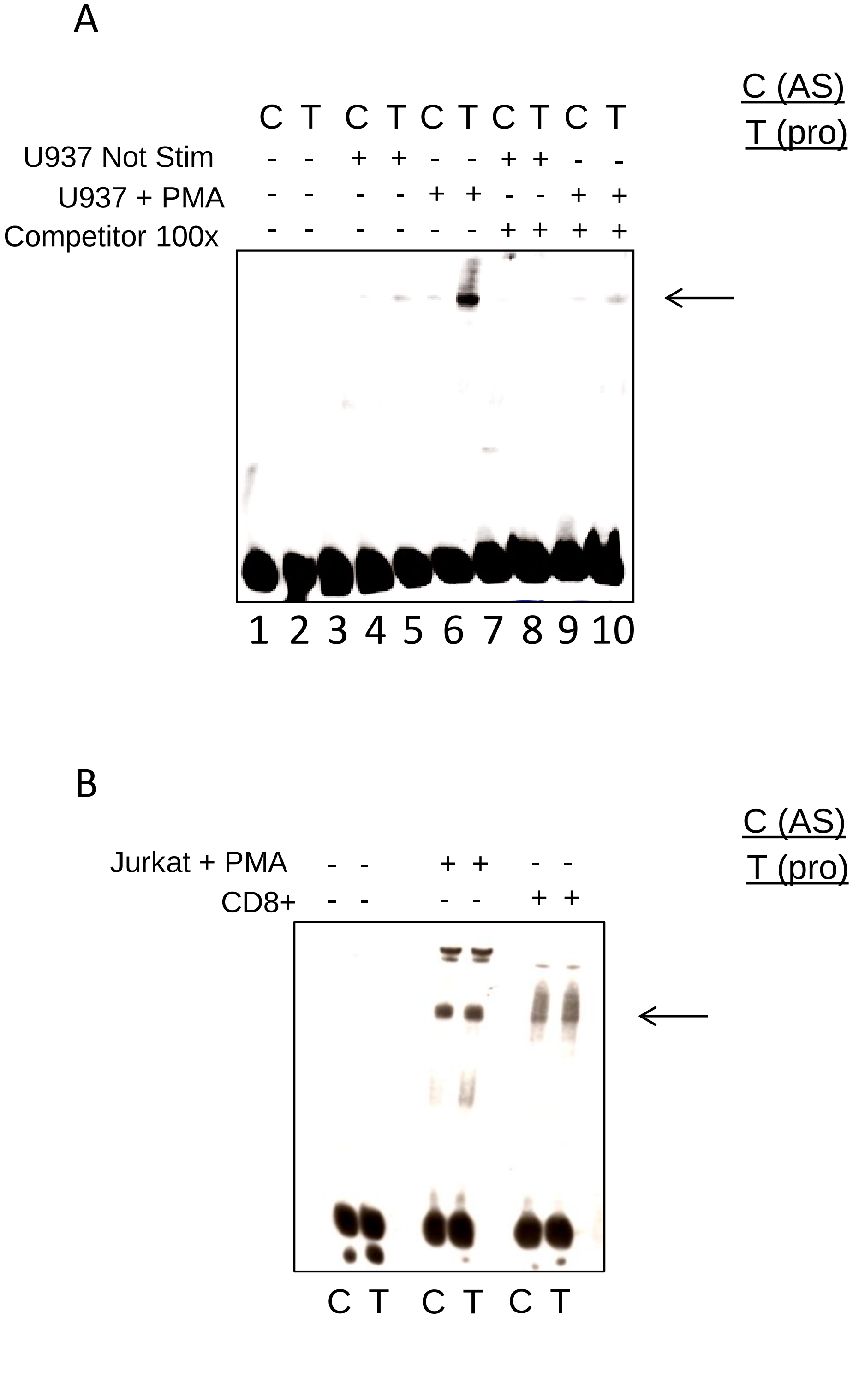

Supplementary Figure 3

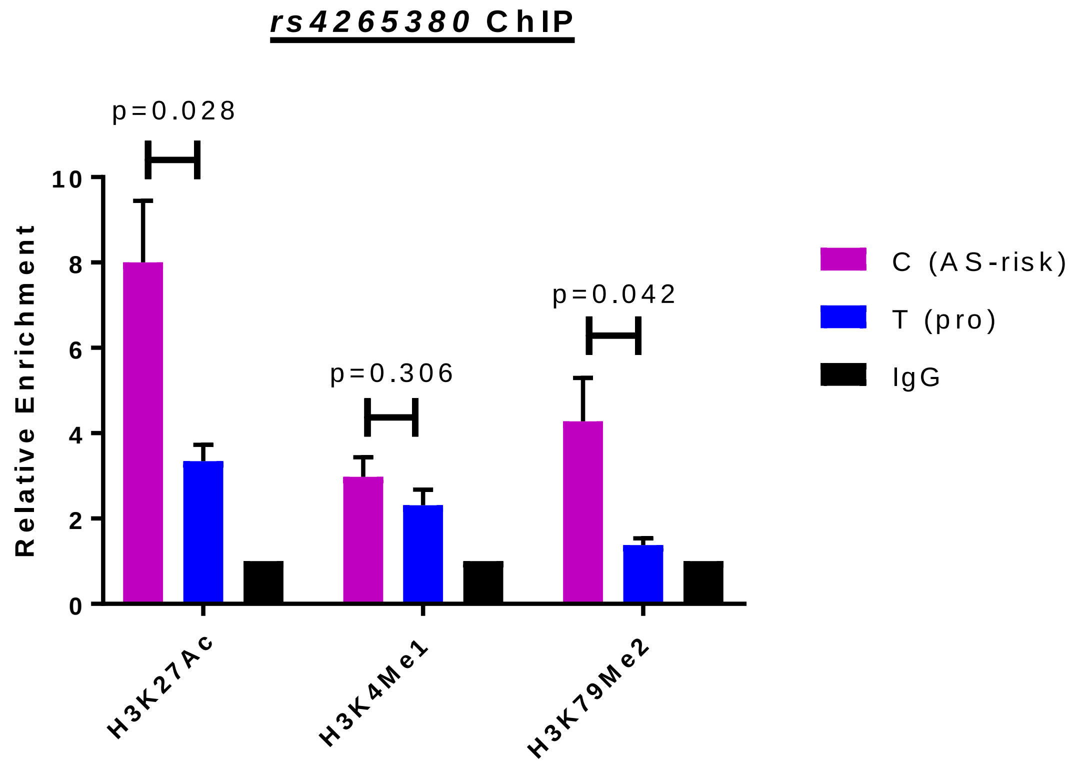

Suppl. Figure 1: RUNX3 mRNA expression is decreased in AS cases independently by *rs4265380* genotype.

Analysis of published RNA-seq experiments [19] show RUNX3 expression on 134 peripheral blood mononuclear samples (72 AS patients and 62 healthy controls). Box plot shows the shape of the distribution, its central value, and its variability. RUNX3 is downregulated in total cases vs total controls (log2foldchange=-0.233, p=0.002).

Suppl. Figure 2: *rs4265380* genotype influences protein/DNA complex formation. (A) Representative EMSA showing U937 nuclear extract/DNA complex formation (see arrow) with enhanced complex formation for the AS-risk allele T (lane 6) after stimulation with PMA. A very faint complex is also present in unstimulated samples (lanes 3 and 4). Competition experiments with 100 fold excess of unlabelled competitor (lanes 7-10) confirm signal specificity**.** (B) *rs4265380* probes were also tested for CD8+ T-cells and Jurkat stimulated with PMA: a representative EMSA is shown.

Suppl. Figure 3. The genomic region spanning *rs4265380* has an effect on H3K79Me2 methylation and H3K27Ac acetylation. Three independent chromatin immunoprecipitation experiments followed by allele-specific qPCR on *rs4265380* CT heterozygote CD14+ monocytes (stimulated for 24 hours with LPS) from AS patients assessed the relative chromatin enrichment H3K4Me1, H3K79Me2 (p=0.042) and H3K27Ac (p=0.028). The fold enrichment is expressed as mean ± SEM for each patient in triplicate. P-values were determined using ANOVA.
